# How are new nurses satisfied with their jobs? From the work value perspective of Generations Y and Z nurses

**DOI:** 10.1186/s12912-024-01928-7

**Published:** 2024-04-20

**Authors:** Eunkyung Kim, Heejung Kim, Taewha Lee

**Affiliations:** 1 College of Nursing, Eulji University, 712 Dongil-Ro, Uijeongbu-Si, Gyeonggi-Do 11759 South Korea; 2 Mo-Im Kim Nursing Research Institute, College of Nursing, Yonsei University, 50-1 Yonsei-Ro, Seodaemun-Gu, Seoul, 03722 South Korea

**Keywords:** Nurses, Generations, Generation Y, Millennials, Generation Z, Work value, Job satisfaction

## Abstract

**Background:**

Job satisfaction has garnered significant interest across multiple disciplines as it plays a vital role in shaping human resource strategies. In the field of nursing, enhancing job satisfaction can help prevent workforce shortages. Work values and job-related characteristics are significant predictors of job satisfaction. However, the influence of factors may change as younger generations join the nursing workforce. Although research on generational commonalities and differences in work values is increasing, there is insufficient information on generational differences in the interplay between work values and job satisfaction. This study investigated the factors associated with job satisfaction of new nurses in each generational group based on a work value perspective.

**Methods:**

A total of 280 new nurses (151 from Generation Y and 129 from Generation Z) were selected from the Graduates Occupational Mobility Survey. Multiple linear regression analyses were performed to determine the factors associated with job satisfaction in both groups.

**Results:**

Most participants graduated with a diploma (61.1%), were paid less than the average salary of each group (60.4%), and conducted shift (72.9%) and overtime work (64.3%). Work values and job satisfaction levels were not significantly different between the two groups. Multiple linear regression analyses showed that career growth and task work values were associated with job satisfaction for Generation Z, while task, reputation, and environment work values were associated with job satisfaction for Generation Y. Among the job-related characteristics, nurses’ job tenure was associated with job satisfaction in both groups; salary and overtime had varying relationships with job satisfaction between the two generations.

**Conclusions:**

Understanding generational differences is crucial for improving the effective management of new generational nurses. Our study findings support that different work value dimensions and job-related characteristics were associated with job satisfaction in each generation. Accordingly, it is essential to develop distinct initiatives, such as a well-structured program, to support the continued career growth of the new Generation Z nurses, thereby enhancing their job satisfaction. Furthermore, providing a conducive working environment that helps new-generation nurses overcome challenges and ensures personal lives should be considered.

## Background

In recent decades, job satisfaction has gained significant attention in various fields, such as management and organizational psychology [1]. Job satisfaction refers to the fulfillment of desired needs within a working environment and gratifying emotional responses toward working conditions [2]. In the nursing field, a recent systematic review found that nurses’ job satisfaction was related to their behaviors, quality of care, and organizational outcomes [3]. For example, previous research has found that Italian nurses with higher job satisfaction were more likely to successfully complete their tasks [4]. A Finnish study showed that nurses’ job satisfaction was positively associated with their patients’ perceived quality of care [5]. Conversely, nurses with lower job satisfaction tend to have a higher intention to leave their hospitals [6] or the nursing profession permanently [6, 7]. Given that new nurses have a high prevalence of turnover [8], enhancing their job satisfaction is vital for retaining the nursing workforce [6]. For this reason, researchers have identified its related factors such as individual factors (e.g., age, education, and marital status), job-related factors (e.g., salary, shift, and overtime demand), and attitudes toward work [1]. Among the identified factors, work value should be considered when developing strategies to increase job satisfaction [9, 10].

Value is a construct involved in evaluating one’s outcomes or activities [11], and work value is formed by considering work in terms of general values [9]. Work value refers to the satisfaction or reward that individuals seek from work [12] and can motivate their behaviors by influencing their attitudes and goals [11]. A growing body of research has shown significant relationships between work value and job satisfaction [10, 13, 14]. For example, higher work value among nurses has been associated with greater job satisfaction in Taiwan [14] and Italy [15].

Work value has conventionally been classified into two types: intrinsic (i.e., interest or satisfaction from the work itself, including personal growth and accomplishing challenging work) and extrinsic (i.e., preference toward external rewards, such as pay, work environment, or recognition) [16]. A considerable amount of literature has treated work values as these two components [13, 15]. However, there is a view that it is necessary to focus on detailed dimensions to fully capture work value [17], and studies that classify work values in various ways have recently been conducted. For instance, one study measured work value using four aspects (i.e., extrinsic/instrumental, intrinsic/cognitive, social/altruistic, and prestige/status) [18], while another study assessed work value using altruism, professional autonomy, professional development, and achievement dimensions [19]. Although such measures would have the advantage of providing a specific understanding of the population, leading to various strategies for improving outcomes, studies investigating the association between specific dimensions of work values and job satisfaction are limited.

The nursing workforce is continuously changing. Recently, nurses from Generation Z, a new arising generation, have entered the workforce, replacing older generations [20]. The concept of a generation refers to a cohort that shares the same birth years and significant societal events, such as world events and technological, economic, and social shifts collectively [21, 22]. Due to these formative experiences, cohorts develop distinctive characteristics that differentiate them across generations [21]. Given that generational theory suggests that although Generation Z (born between 1995 and 2012) shares many commonalities with Generation Y (born between 1980 and 1994), this generation possesses distinct characteristics [23]. For example, Generation Z tends to prioritize work-life balance and enjoyable work, whereas Generation Y assigns higher importance to careers and success and focuses more on the work itself [24, 25]. Moreover, Generation Z has a greater preference for working independently over working in a team and prefers to receive continuous feedback on their performance compared with previous generations [26]. In other words, each generation has different views, attitudes, and work-related expectations; accordingly, job-related characteristics influencing job satisfaction, such as job tenure, hospital region, salary, shift, and overtime work may vary across generations [27–31]. This implies that if managers fail to grasp the distinctions among generations in terms of how they fulfill job-related needs and preferences, it can lead to a decline in both job satisfaction and productivity [21]. Additionally, a previous study demonstrated that work values differ across multiple generations due to cohort effects [32]. While numerous previous research has explored generational differences and similarities in work values [33, 34] and each generation’s perception toward work [18, 35], the majority of such studies have struggled to distinguish differences arising from generational cohorts and those attributed to age because of the nature of cross-sectional data [36]. Moreover, evidence of the intergenerational difference in the associated factors of job satisfaction is lacking, especially from the work value perspective. Previous studies have reported that understanding generational differences in work values would help develop strategies and policies to motivate employees and satisfy their needs across different generations [11, 37].

Given the differences between Generations Y and Z, who comprise a significant portion of the current nursing workforce, a better understanding of what contributes to job satisfaction for younger generations will help management develop effective strategies to retain these nurses. To address the aforementioned gap, this study aimed to determine which dimensions of work values and job-related characteristics were associated with job satisfaction among new Generations Y and Z nurses in South Korea.

## Method

### Design

This correlational study used a secondary data analysis of the national data from the Graduates Occupational Mobility Survey (GOMS).

### Description of GOMS data

The GOMS is an annually conducted nationally representative survey of college graduates, meticulously quality-controlled by the Korean Employment Information Service [38]. The purpose of the survey is to provide fundamental data for the establishment of policies to decrease the disparity between education and the labor market by investigating career development and job transfer paths for college graduates. The GOMS has 18 domains including college graduates' educational curriculum, job search and experience, vocational training, and the transition from school to the labor market. The survey participants were graduates who had completed an associate’s degree or higher in the previous year. The interviewers visited graduates who agreed to participate, asked questions, and recorded their responses. The data were collected from September 1st to November 30th each year and released in February of the following year.

### Sampling for the secondary data analysis

**Fig. 1:**
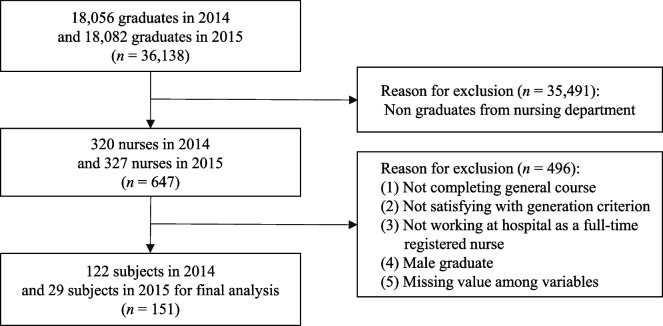
Flowchart of samples for Generation Y

**Fig. 2:**
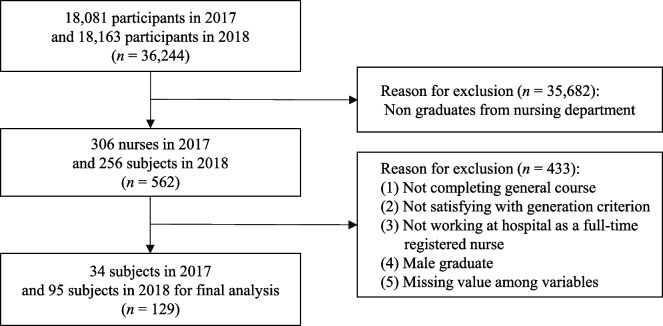
Flowchart of samples for Generation Z

The data in the present study were derived from the 2014–2015 and 2017–2018 GOMS. The study samples for this secondary data analysis were Generation Y or Z registered nurses working full-time in hospitals. Although varying opinions exist regarding the exact time frames defining generational cohorts [36], in this study, Generation Y was defined as those born between 1990 and 1991, and Generation Z as those born between 1995 and 1996. This comparison aimed to explore relatively close generational groups in the context of rapid social changes during recent decades [22] and to contribute to the sensitive adaptation of organizations to emerging generations. Furthermore, these operational definitions of Generations Y and Z were determined based on: (a) the definitions of birth years of Generations Y and Z by researchers [39, 40]; and (b) the utilization of common survey questions with slight variations in survey items each year. The exclusion criteria were nurses who (a) were male; (b) completed a transfer program for a Bachelor of Science in Nursing (BSN) degree; (c) were working at public agencies, clinics, or research agencies; and (d) had any missing data on key variables. Male nurses were excluded because most were required to complete two years of military service during their college years in South Korea. Due to this military service, male nurses exhibit different patterns in interpersonal relationships, organizational adaption, and school-life adjustment compared to other students [41, 42]. The analyses included 280 new nurses, 151 from Generation Y, and 129 from Generation Z (Figs. 1 and 2).

### Measures

#### Job satisfaction

Job satisfaction was measured with the question, “How satisfied are you with each aspect of your current job?” and a total of 13 items (e.g., quality of work environment, work autonomy, and promotion systems) were included, which have been used to assess job satisfaction in previous research [43]. All items were rated on a 5-point Likert scale (1 = strongly dissatisfied; 5 = strongly satisfied). In this study, the Cronbach’s alpha for this measure was 0.89.

#### Work value

Work value was determined with the question, “How important is each aspect when choosing the work?” and these 15 items were classified into four dimensions according to a previous study [44]. The dimensions were as follows: (a) career growth (four items: own aptitude and interest, personal development potential, job prospects, and employment stability), (b) task (three items: relevance to the major, task difficulty, and workload), (c) reputation (three items: workplace size, social reputation for a job, and social reputation for work), and (d) environment (five items: salary, working time, work environment, welfare benefits system, and commuting distance). All items were rated on a 5-point Likert scale (1 = strongly not important; 5 = strongly important), and mean scores were calculated for each dimension. McDonald’s omega is widely regarded as a robust and accurate estimator of reliability due to its reduced reliance on stringent statistical assumptions [45]. It also proves beneficial for evaluating the formative [46] and multidimensional constructs [47]. In this study, the McDonald’s omega for reputation was 0.84, career growth 0.73, task 0.74, and environment 0.70.

#### Individual and job-related characteristics

The individual characteristics included age and school type. School type was dichotomized into (a) diplomas and (b) BSN. The job-related characteristics included job tenure, hospital region, salary, shift work, and overtime. Job tenure was classified as (a) 12 months or less and (b) 13 months or more. The hospital region had two options: capital and non-capital. Salary was dichotomized into (a) average or above and (b) below average based on the average salary of each generational group according to self-reports. Those who answered “yes” to the question “Are you a shift worker?” were classified as shift workers, and the others were classified as non-shift workers. Overtime status was assessed using self-reports of weekly average hours for overtime work, and respondents with one or more hours were categorized as having worked overtime.

### Data analysis

Data were analyzed using IBM SPSS Version 26.0 (SPSS Corp., College Station, TX, USA), with the two-tailed significance level set at 0.05. Descriptive statistics were used to characterize participants. Two separate multiple linear regression analyses were conducted to determine how work value dimensions and job-related characteristics were associated with job satisfaction in each generational group. When examining the data distribution, it was confirmed that normality was satisfied; common methods variance analysis and outlier testing revealed no identifiable problems. For collinearity diagnosis, the tolerance, variance inflation factor (VIF), and Durbin-Watson statistics were examined. The tolerance ranged from 0.61 to 0.96 for Generation Y and 0.57 to 0.92 for Generation Z, that is, lower than 1. The values of VIF were 1.04–1.65 for Generation Y and 1.09–1.75 for Generation Z, lower than 10, and Durbin-Watson statistics were 1.45 for Generation Y and 1.26 for Generation Z; therefore, issues regarding multi-collinearity and autocorrelation were not found for this study.

### Ethical considerations

The collection of GOMS data followed the acquisition of participant consent in accordance with the Declaration of Helsinki. Since the anonymized data is openly available for scientific purposes, the Institutional Review Board of Eulji University Health System waived the necessity for informed consent for this study (Approval No: EUIRB2023-050).

## Results

### Participants’ characteristics

The mean age of nurses (*N* = 280) was 24.26 ± 0.64 years, and more than half had completed diploma degrees. Most had worked for 13 months or more at a hospital in a non-capital area. Most participants also received a salary below average for each generation and engaged in shift and overtime work. When comparing two generational groups, Generation Y (*n* = 151) was statistically older than Generation Z (*n* = 129) (24.53 vs. 23.93, *p* < 0.001). Compared to Generation Y, Generation Z was more likely to complete BSN courses (32.5% vs. 46.5%, *p* = 0.016), receive a salary with an average of or more than their generation group (29.8% vs. 51.2%, *p* < 0.001), engage in shift work (62.9% vs. 84.5%, *p* < 0.001), and have less overtime work (70.9% vs. 56.6%, *p* = 0.013) (Table 1).

**Table 1:** Comparisons between generational groups of individual and job-related characteristics (*N* = 280)

Variables		Total (*N* = 280)	Generation Y (*n* = 151)	Generation Z (*n* = 129)	*t* or χ^2^	*p-*value
		M ± *SD* or *n* (%)				
Individual characteristics						
Age (year)		24.26 ± 0.64	24.53 ± 0.66	23.93 ± 0.45	8.963	< 0.001
School type	Diploma	171 (61.1)	102 (67.5)	69 (53.5)	5.786	0.016
	BSN	109 (38.9)	49 (32.5)	60 (46.5)		
Job-related characteristics						
Job tenure (month)	12 or less	89 (31.8)	53 (35.1)	36 (27.9)	1.660	0.198
	13 or more	191 (68.2)	98 (64.9)	93 (72.1)		
Hospital region	Capital	75 (26.8)	42 (27.8)	33 (25.6)	0.177	0.674
	Non-capital	205 (73.2)	109 (72.2)	96 (74.4)		
Salary	Average or above	111 (39.6)	45 (29.8)	66 (51.2)	13.267	< 0.001
	Below average	169 (60.4)	106 (70.2)	63 (48.8)		
Shift work	Yes	204 (72.9)	95 (62.9)	109 (84.5)	16.386	< 0.001
	No	76 (27.1)	56 (37.1)	20 (15.5)		
Overtime work	Yes	180 (64.3)	107 (70.9)	73 (56.6)	6.172	0.013
	No	100 (35.7)	44 (29.1)	56 (43.4)		
Work value	Career growth value	4.19 ± 0.52	4.24 ± 0.53	4.14 ± 0.51	1.650	0.100
	Task value	4.05 ± 0.56	4.04 ± 0.57	4.07 ± 0.54	-0.477	0.634
	Reputation value	3.77 ± 0.70	3.78 ± 0.69	3.76 ± 0.70	0.145	0.885
	Environment value	4.32 ± 0.43	4.30 ± 0.42	4.36 ± 0.44	-1.182	0.238
Job satisfaction		3.40 ± 0.58	3.39 ± 0.58	3.42 ± 0.59	-0.387	0.699

*BSN* Bachelor of Science in Nursing,* M* Mean, *SD* Standard deviation

### Levels of job satisfaction and the work value of new Generations Y and Z nurses

The results showed that environment value was the most important work value dimension for both generational groups, followed by career growth, task, and reputation. Generation Y nurses exhibited higher levels of career growth (4.24 vs. 4.14, *p* = 0.100) and reputation (3.78 vs. 3.76, *p* = 0.885) in contrast to Generation Z, while task (4.04 vs. 4.07, *p* = 0.634) and environment (4.30 vs. 4.36, *p* = 0.238) values were comparatively lower; however, no significant differences were detected between these two groups. When comparing job satisfaction levels between Generations Y and Z nurses, although the average for Generation Z nurses was higher than that of Generation Y nurses, it was also not statistically different (*t* = -0.387, *p* = 0.699) (Table 1).

### Factors associated with job satisfaction in Generations Y and Z

The multiple regression model for Generation Y explained a 22.9% variance in job satisfaction and a 12.4% variance for Generation Z. Among work value dimensions, career growth value (β = 0.301, *p* = 0.003) was associated with job satisfaction in Generation Z; while reputation (β = 0.343, *p* < 0.001) and environment value (β = -0.292, *p* = 0.001) were significant in Generation Y. Task value was a common factor of job satisfaction in both Generations Y and Z. It was positively associated with job satisfaction in Generation Y (β = 0.188, *p* = 0.031); however, a negative association between task value and job satisfaction was revealed in Generation Z (β = -0.195, *p* = 0.044). Among job-related characteristics, job tenure was identified as a related factor of job satisfaction in both generational groups (β = -0.223, *p* = 0.003 for Generation Y, β = -0.187, *p* = 0.038 for Generation Z). Salary (β = 0.215, *p* = 0.013) was associated with Generation Y’s job satisfaction, and overtime work (β = -0.176, *p* = 0.043) was associated with Generation Z’s job satisfaction (Table 2).

**Table 2:** Factors associated with job satisfaction in Generations Y and Z (*N* = 280)

Variables	Generation Y				Generation Z			
	b	SE	β	*t*	b	SE	β	*t*
Job tenure (ref: 12 or less)	-0.312	0.104	-0.223	-3.011^**^	-0.283	0.135	-0.187	-2.097^*^
Hospital region (ref: non-capital)	-0.234	0.128	-0.157	-1.823	0.081	0.146	0.052	0.553
Salary (ref: below average)	0.314	0.125	0.215	2.511^*^	0.126	0.129	0.092	0.978
Shift work (ref: no)	-0.038	0.101	-0.027	-0.374	-0.210	0.166	-0.112	-1.263
Overtime work (ref: no)	-0.139	0.108	-0.094	-1.292	-0.242	0.118	-0.176	-2.042^*^
Career growth value	0.124	0.117	0.098	1.064	0.404	0.135	0.301	2.981^**^
Task value	0.222	0.102	0.188	2.181^*^	-0.244	0.120	-0.195	-2.037^*^
Reputation value	0.331	0.084	0.343	3.952^***^	0.074	0.095	0.076	0.770
Environment value	-0.462	0.140	-0.292	-3.290^**^	-0.033	0.169	-0.021	-0.195
Model fit	*R*^2^ = 0.276, Adjusted *R*^2^ = 0.229, *F* = 5.962^***^				*R*^2^ = 0.185, Adjusted *R*^2^ = 0.124, *F* = 3.007^**^			

*b* Unstandardized regression coefficient, *SE* Standard error, *β* Standardized regression coefficient

^*^p < 0.05, ^**^p < .01, ^***^p < .001

## Discussion

This study identified the factors associated with job satisfaction for Generations Y and Z. Among the work value dimensions, career growth, and task values were associated with job satisfaction for Generation Z, while reputation, task, and environment values were factors of job satisfaction for Generation Y. Additionally, job tenure was a common factor of job satisfaction in both generational groups; salary and overtime work were differentially associated with job satisfaction in each group. This study contributes to a deeper understanding of what is associated with job satisfaction of new-generation nurses compared to a previous generational cohort.

Our study findings confirmed that there are no significant differences in work values between Generations Y and Z. This is similar to existing evidence which reports that empirical differences between generations are generally very limited due to exceeding within-group differences [36]. This phenomenon might be attributed to the fact that the social identity of the generation is shaped by both shared experiences (i.e., commonality) and the uniqueness of each member (i.e., heterogeneity) [48]. Furthermore, in the context of historical, technological, behavioral, and attitudinal data revealing a continuum across generations rather than a distinct threshold [22], rising generations are influenced by and reflective of previous generations [21]. This study also affirmed the prioritization of work values within Generations Y and Z, wherein the hierarchical order was as follows: environment, career growth, task, and reputation values. These findings align with trends observed in prior studies in the nursing field indicating that Generation Z nurses, who represent an emerging generation, place more value on tangible rewards, such as pay and benefits, than work itself and prestige [18, 49]. However, this finding differs from Indonesian evidence on Generation Z employees at the beginning of their careers, which indicates that the top work value was self-development through jobs, followed by salary or social appreciation [50]. Additionally, it was revealed that intrinsic values such as sustainable growth, were of utmost priority beyond extrinsic aspects (e.g., promotion and salary) [51]. These gaps across occupational fields may reflect nurses’ poor work environment, such as overtime and shift work [52] since work values reflect individuals’ awareness of what they want to accomplish in their work [53]. Although the current study contributes to the understanding of the new generations, more research should be conducted to gain deeper insights into these generations’ work attitudes through comparisons across occupations.

Notably, although the level of each work value dimension was not significantly different between the two groups, there were differences in the work value dimensions that contributed to job satisfaction for each group. Reputation and environment values, related factors of Generation Y’s job satisfaction, can be categorized as extrinsic work value, whereas career growth value, a factor of Generation Z’s job satisfaction, can be categorized as an intrinsic aspect [44]. Based on this classification, our findings are similar to existing evidence. For example, a Chinese study of Generation Y hotel employees found that extrinsic work values regarding being well-paid or good working conditions had greater effects on job satisfaction than intrinsic aspects [54]. Moreover, a systematic review comparing perceptions of job satisfaction between Generations Y and Z revealed that Generation Y perceived job satisfaction as having adequate opportunities for promotion and well-paid wages based on their work experience and performance [55], which are extrinsic aspects of work value [56]. Dissimilar to Generation Y’s perception, job satisfaction for Generation Z indicates personal growth in skill and knowledge, that is, intrinsic work value [56]. Furthermore, Generation Z employees expect opportunities for professional development and growth in the workplace, distinct from other generations [57]. Hence, to promote job satisfaction among new-generation nurses, this study’s findings highlight that it is crucial to establish initiatives that consider the differences in the dimensions of work values associated with job satisfaction. To address the career growth value of Generation Z nurses, effective strategies such as residency programs for promoting the continued growth of clinical knowledge, communication skills, and professional development in the clinical context are necessary to encourage them to seek career growth value in the workplace [58].

Interestingly, task value was associated with job satisfaction in both generational groups; however, it was positively related to job satisfaction for Generation Y and negatively related to job satisfaction for Generation Z. This study revealed that the trend of the relationship between task value and job satisfaction has changed. Task value was relevant to the importance of task difficulty and workload when choosing a job; therefore, performing challenging tasks and working hard might have positive effects on job satisfaction for Generation Y but not for Generation Z. This could be related to the distinct attitudes toward work between Generations Y and Z. A previous study found that Generation Y perceives work as meaningful and considers taking on new challenges as important to their individual careers [35]. However, Generation Z does not consider taking on challenging tasks or hard work relative to their abilities as important [18]. Moreover, they look for an enjoyable workplace [26] and are satisfied with their job when it is viewed as interesting [56]. Although comparative research on attitudes toward work between Generations Y and Z is limited, most prior studies have reported that the more recent generations tend to be less interested in hard-working [59]. Considering that many new nurses have achieved clinical competencies in numerous challenging and stressful situations [60–62], this study suggests that new nurses in Generation Y feel fulfilled by working hard with challenging tasks, which increases their job satisfaction, whereas Generation Z has a negative perception of challenges that deviate from their work expectations (i.e., work should be interesting), showing a negative association between their task value and job satisfaction. Thus, new-generation nurses may require enhanced organizational support, such as mentorship, to help them overcome difficulties and hardships [60]. Moreover, a friendly working environment that provides tangible support and feedback [63] can facilitate new nurses’ perceptions of a workplace and lead them to consider it as a good place to work. Such managerial efforts should be considered to promote job satisfaction among new generations.

Among the various job-related characteristics, our findings revealed that job tenure was a common factor of job satisfaction in both generational groups and that nurses with more than 12 months of employment were more likely to have a lower level of job satisfaction. These results are supported by prior research showing that new nurses’ job satisfaction decreases as their working experience increases [27, 64]. A previous study found that a significant number of new nurses still lack confidence in their skills and feel uncomfortable even after 12 months [65]; however, the majority of intensive programmed support for new nurses has been provided for less than the first 12 months [66]. Moreover, although the new generation values personal attention, feedback, and information [26], they perceive that the amount of feedback and information they receive diminishes over time [27]. Such insufficient timed support would negatively affect the job satisfaction of new nurses with more than 12 months of job tenure. Therefore, nurse leaders and senior nurses should consistently offer support, provide feedback on work performance, and keep nurses updated on workplace-related information [67]. This helps cultivate a sense of respect and value among nurses during their early careers [68, 69], ultimately contributing to enhanced job satisfaction.

Additionally, salary was significantly associated with job satisfaction for Generation Y and overtime status for Generation Z. These findings are supported by previous research showing that the recent generation is more likely to be central to their personal lives and value leisure more than the older generation [59, 70]. Moreover, our findings were similar to those of a previous study demonstrating that the newer generation was more dissatisfied with their lack of personal life and overtime than the older cohort [71]. As evidenced by prior research findings, it can be inferred that the newer generations value personal time more. A recent study on Generation Y nurses in New Zealand revealed that the most desired change in the nursing field was related to salary, prioritized over personal time [72], indicating a tendency to place greater emphasis on monetary compensation. Generation Z prioritizes flexible working schedules and paid vacation over salary when choosing a job [73, 74], suggesting that a healthy work-life balance is crucial for job decisions [57, 75]. Over an extended period, the growing disparity between the demand and supply of nurses has given rise to a worldwide issue [76]. To ensure a sufficient nursing workforce, organizations are increasingly prioritizing the proactive enhancement of work-life balance and adopting greater flexibility in working hours [77]. This is to respond to the demands of new generations entering the workforce who advocate for a fluid and flexible work environment [75]. With such labor market changes, the status of overtime work among Generation Z nurses might influence the result of this study. Therefore, this study’s noteworthy findings suggest establishing systematic support to tackle overtime work and ensure the personal time and lives of new generation nursing staff.

### Limitation

Our study has several limitations. First, due to the nature of secondary data analysis, we could not include a few variables that are known predictors of nurses’ job satisfaction, such as work environment (e.g., patient assignments, and working schedule). Additionally, salary was assessed based on subjective self-report data. Second, two variables, job satisfaction, and work values, were assessed using items developed through experts’ meetings for GOMS. It is advisable to employ instruments with high validity to measure these constructs in future research. Moreover, given that measures for work values appear formative, suggesting that the items collectively define the concept of work values rather than reflecting an existing construct [78], this aspect deserves careful consideration when interpreting our study’s findings. Future studies should carefully investigate the measurement properties of work values and explore potential variations in the relationship between work values and job satisfaction. This will help enhance the understanding of work values and their implications for job satisfaction. Third, the current study exclusively focused on female Korean nurses; therefore, the findings might be limited in their generalizability to female nurses only. Since work value could differ across nations [48], sex [32], or occupational fields [79], further studies are required to fully understand the associations between work value and job satisfaction for new-generation nurses. Finally, since this was a cross-sectional study, inferring causation between work values and job satisfaction and controlling for variations that might arise during individuals’ development or labor market changes were not feasible. Despite these limitations, this study used a nationally representative dataset to compare two generational groups of similar ages (i.e., time-lag research). Given previous literature reporting that work values are influenced more by generation than by age or maturity [80], the current study provides a greater understanding of how work values contribute to job satisfaction according to generation.

## Conclusion

The influx of Generation Z into the workplace has resulted in a remarkable change in the workforce. Generational differences in work values and attitudes contribute to the complexity of the work environment and present challenges for nursing leaders and administrators in maintaining a stable workforce. Therefore, it has become more important to understand the new generation’s unique characteristics, and nurse leaders and organizations should prioritize successful initiatives and policies for this workforce. Thus, previous generations, including Generation Y, should understand the notable aspects of Generation Z to effectively manage them using distinct human resource management strategies. The results of this study provide evidence that work value dimensions are differentially associated with job satisfaction across generations. Hence, nursing leaders should establish a structured support program for new Generation Z nurses to fulfill their career growth value and provide a good working environment to help these nurses overcome challenging tasks. Additionally, it is essential to ensure personal time while minimizing overtime to improve Generation Z’s job satisfaction.

## Data Availability

The data supporting the findings of this study are available to the public at: https://survey.keis.or.kr/goms/gomsdownload/List.jsp.

## Abbreviations

BSN: Bachelor of Science in Nursing
GOMS: Graduates Occupational Mobility Survey
M: Mean
SD: Standard deviation
SE: Standard error
VIF: Variance inflation factor
